# All-Semiconductor Plasmonic Resonator for Surface-Enhanced Infrared Absorption Spectroscopy

**DOI:** 10.3390/mi8010006

**Published:** 2017-01-19

**Authors:** Wei Wei, Jinpeng Nong, Xiao Jiang, Na Chen, Suqin Luo, Linlong Tang

**Affiliations:** 1 Key Laboratory of Optoelectronic Technology & Systems, Ministry of Education of China, College of Optoelectronic Engineering, Chongqing University, Chongqing 400044, China; nongjp@cqu.edu.cn (J.N.); 20103279@cqu.edu.cn (X.J.); 20150802007@cqu.edu.cn (N.C.); 2 Chongqing Research Center for Advanced Materials, Chongqing Academy of Science and Technology, Chongqing 401123, China; luosuqin@cqu.edu.cn; 3 Chongqing Key Laboratory of Multi-scale Manufacturing Technology, Chongqing Institute of Green and Intelligent Technology, Chinese Academy of Sciences, Chongqing 401122, China; tll@cigit.ac.cn

**Keywords:** SEIRAS, semiconductor, plasmonic, resonator, mid-IR

## Abstract

Infrared absorption spectroscopy remains a challenge due to the weak light-matter interaction between micron-wavelengthed infrared light and nano-sized molecules. A highly doped semiconductor supports intrinsic plasmon modes at infrared frequencies, and is compatible with the current epitaxial growth processing, which makes it promising for various applications. Here, we propose an all-semiconductor plasmonic resonator to enhance the infrared absorption of the adsorbed molecules. An optical model is employed to investigate the effect of structural parameters on the spectral features of the resonator and the enhanced infrared absorption characteristics are further discussed. When a molecular layer is deposited upon the resonator, the weak molecular absorption signal can be significantly enhanced. A high enhancement factor of 470 can be achieved once the resonance wavelength of the resonator is overlapped with the desired vibrational mode of the molecules. Our study offers a promising approach to engineering semiconductor optics devices for mid-infrared sensing applications.

## 1. Introduction

Infrared (IR) spectroscopy of the distinct vibrational and rotational molecular resonances provides a powerful tool for the analysis and characterization of a wide range of molecules [1,2,3]. However, the wavelength of light required to excite these resonances is often orders of magnitude larger than the absorption cross-sections of the molecules. This mismatch makes infrared detection and identification of nanoscale volumes of molecules challenging. Plasmonic enhancement is one of the most effective approaches to improve the light-matter interaction [4,5,6,7,8,9], which results in the emergence of surface-enhanced infrared absorption spectroscopy (SEIRAS) and recieved intense attention due to the possibility of single-molecule detection. It has been demonstrated that localized surface plasmonic polarization (LSPP) can be excited in the noble nanostructured metals, which can concentrate the electric fields in the near-field of the metal [5,6], whereas the resonance wavelength of these structures mainly locates at the near-IR and visible frequencies. When the wavelength is expanded into the mid-IR region, the permittivity of the metals become increasingly large, and the metals begin to more closely approximate a perfect electrical conductor, which leads to the weak confinement of electric fields on the metallic surfaces. Alternatively, though metallic microantennas [7,8,9] have subsequently been used to couple mid-IR light to adsorbed molecules, it requires antennas with a length on the wavelength scale, which imposes very real limitations on the antenna array density and the achievable field enhancement.

Plasmonic behavior at the mid-IR wavelength range has been observed in highly doped semiconductors, such as doped silicon [10,11], germanium [12,13], GaAs [14,15], as well as InAs [16,17], which provides an opportunity to address this challenge. The high doping concentrations of the semiconductor result in a plasma frequency in the infrared region, which can be tuned by adjusting the doping density [18,19]. Therefore, highly doped semiconductors can mimic, at long wavelengths, the optical properties of traditional plasmonic metals at shorter wavelengths. In addition, the semiconductor is mass-producible and compatible with the current epitaxial growth processing [19], which makes it promising for various applications. Recently, Joshua A. Mason reported an absorber based on a semiconductor plasmonic structure for mid-infrared sensing using metal as the reflective layer [20]. The sensing medium is embedded in the absorber structure. However, its application is limited since the nanoscale gap is relatively difficult to construct between the plasmonic structure and the Au layer. More importantly, there is an intrinsic incompatibility of low-quality metal deposition with high-quality epitaxial growth of semiconductors from a practical point of view [19]. Consequently, many intrinsic plasmonic properties can be masked by the poor metal quality or poor semiconductor-metal interfaces. To overcome this problem, an all-semiconductor nanoantenna array utilizing epitaxially grown InAs had been demonstrated for infrared sensing [21]. The weak absorption signal of the molecular layer can be enhanced and observed when the vibrational mode of molecules matches the antenna resonance. Nonetheless, the observed effect of the absorbing molecules is weakly induced by factors including etch-induced material damage, the nanoantenna size, and the overall geometry.

Recently, S. Law et al., proposed an all-semiconductor negative-index plasmonic absorber. Strong absorption can be achieved by coupling into the negative-index, highly confined surface plasmon polariton modes in a multilayer epitaxial structure [22]. In this work, we proposed an all-semiconductor plasmonic resonator with Fabry-Perot–like resonance to enhance the infrared absorption of the molecules. The considered resonator consists of a patterned top layer (highly doped InAs), a spacer layer (undoped InAs) and a bottom layer (highly doped InAs) on the substrate, which can be realized on a chip in a single epitaxial growth process in practice. An optical model is built to systematically investigate the effect of the structural parameters on the optical characteristics of the resonator, including the period, width, and height of the patterned top layer and the thickness of the spacer layer. The enhanced infrared absorption characteristics are further discussed for SEIRAS.

## 2. Structure and Modeling

**Figure 1:**
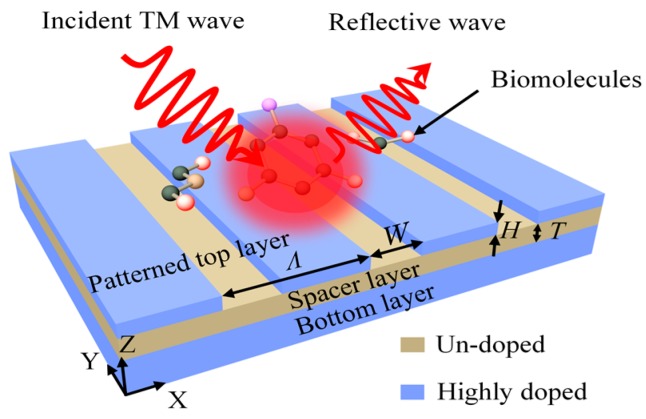
Schematic of the all-semiconductor plasmonic resonator for infrared sensing.

A schematic view of the considered all-semiconductor plasmonic resonator is illustrated in Figure 1. It consists of a patterned top layer (highly doped InAs), a spacer layer (undoped InAs) and a bottom layer (highly doped InAs) on the substrate. In practice, the resonator can be fabricated from a film grown by molecular beam epitaxy on a semi-insulating GaAs substrate. The film consists of an undoped InAs buffer layer followed by a highly doped InAs ground plane, an undoped InAs spacer layer, and a highly-doped InAs top layer. The top layer is then patterned into 1D arrays of stripes with varying stripe widths, periods, and etch depths using standard photolithographic techniques and a wet chemical etch.

To obtain the resonance characteristics of the plasmonic resonator, a physical model is built using COMSOL multiphysics (COMSOL, Stockholm, Sweden) employing the finite element method. The patterned nanostrips have the period of *Λ*, the width of *W*, the height of *H*, and the space thickness between the top layer and bottom layer is *T*. The reflective index of the undoped InAs spacer layer is *n* = 3.5. The optical response of free carriers in a doped semiconductor can be modeled by the Drude formalism as Equation (1) [21,23]:

(1) $$\varepsilon_{m}(\omega )=\varepsilon_{s}\left(1-\frac{{\omega_{p}}^{2}}{\omega (\omega -i\gamma )}\right)$$

where *ε_s_* is the background semiconductor permittivity, *γ* is the free carrier scattering rate, and *ω_p_* is the plasmon frequency given by Equation (2)

(2) $$\omega_{p}=\sqrt{\frac{Ne^{2}}{\varepsilon_{0}\varepsilon_{s}m}}$$

where *N* is the free electron concentration, *e* is the electron charge unit, *ε_0_* is the permittivity in vacuum, and *m* is the effective mass of electrons in the semiconductor. Here, the free carrier scattering rate and plasma frequency are fitting parameters of the highly doped InAs that were extracted from the experimental results in Reference [21].

To simulate the absorption of molecules, a molecular layer with a thickness of 10 nm is assumed to cover on the surface of the resonator. The macroscopic dielectric constant of the molecular layer can be described as a Lorentz-type dispersion in Equation (3) [24,25].

(3) $$\varepsilon (\omega )=\varepsilon_{\infty }+\frac{e^{2}N_{m}/(\varepsilon_{0}m_{e})}{\omega_{m}^{2}-\omega^{2}-i\gamma_{m}\omega }$$

where *ε_∞_* is the dielectric constant of the system at high frequencies, *m_e_* is the electron rest mass, *N_m_* is the volume density of the molecular dipoles, *ω_m_* is the molecular resonance frequency, *γ_m_* is the molecular damping. When the plasmon mode of the resonator is overlapped with the vibrational mode of the molecules, the field confinement of the plasmonic resonance enhances the interaction between the incident light and molecules, resulting in an enhanced absorption signal of the molecules.

## 3. Results and Discussion

### 3.1. The Spectral Features of the Resonator

**Figure 2:**
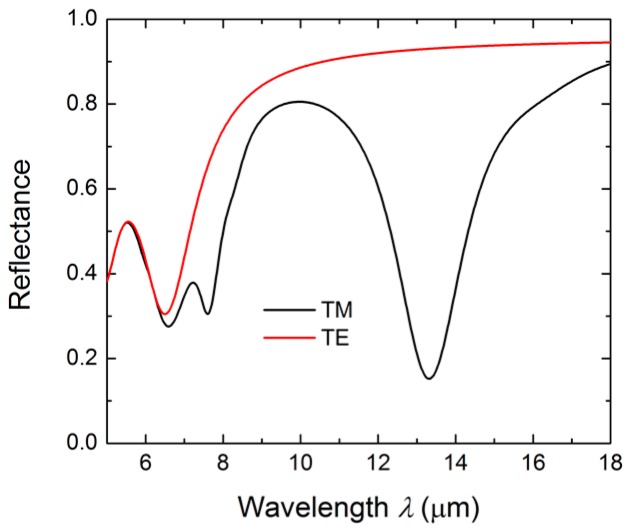
The simulated reflection spectra of the resonator under the transverse-magnetic (TM) and transverse-electric (TE) illumination.

Figure 2 compares the reflection spectra of the resonator under transverse-magnetic (TM) and transverse-electric (TE) illumination, respectively, when *Λ* = 1000 nm, *W* = 500 nm, *H* = 100 nm, and *T* = 200 nm. Obviously, in the considered molecular fingerprint region (6~16 μm), a sharp dip is observed at 13.1 μm in the TM-polarized spectra. However, no such feature is observed in the TE-polarized reflection spectra, indicating that the resonance mode is no longer excited for the TE polarization, and hence there is no enhancement when the resonator is covered with a molecular film. In practice, we can use the built-in control of the spectroscopy to rotate the excitation field polarization to selectively enhance the absorption of the molecular film.

**Figure 3:**
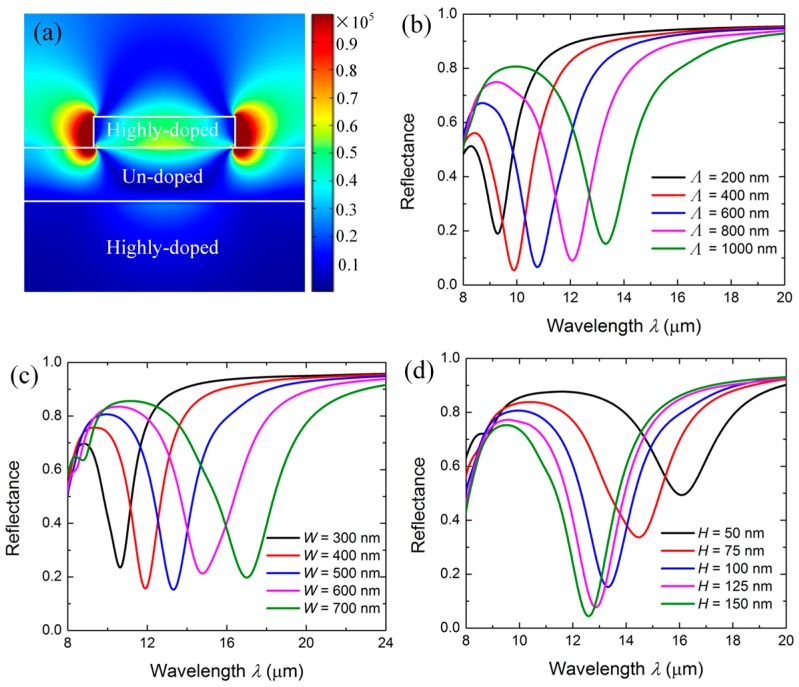
(**a**) Electric intensity profile of the resonator. (**b**) Simulated reflection spectra with various periods of the patterned top layer. (**c**) Simulated reflection spectra with various widths of the patterned top layer. (**d**) Simulated reflection spectra with various heights of the patterned top layer.

The corresponding excited resonance mode profile of the highly doped InAs for TM-polarized light is illustrated in Figure 3a. A localized resonance mode can be obviously observed around the InAs nanostrip, which arises from the excitation and the coupling of the LSPP mode in InAs nanostrips [7,26]. Such a resonance mode exhibits a strong ability to capture light from free space and concentrate optical energy into sub-wavelength spots in the near-field of the InAs strips, resulting in the dramatic enhancement of the peak intensity of the localized electric field |Ex| up to ~1 × 10^5^ V/m, and thus a strong light-matter interaction. This indicates that the resonance dip for the TM polarization in Figure 2 originated from the excited LSPP resonance in the semiconductor plasmonic structure. The spectral features of this resonance peak are mainly determined by the structural parameters of the doped InAs strips and the spacer thickness of the undoped InAs.

We first investigated the effect of the structural parameters of the patterned InAs strips on the spectral features of the resonator. The reflection spectra of the resonator with varying InAs strips period *Λ* are illustrated in Figure 3b when *W* = 500 nm, *H* = 100 nm, and *T* = 200 nm. Each reflection dip represents the plasmonic resonance of the resonators with different *Λ* satisfying the phase matching condition. The resonant wavelength experienced a redshift from 9.3 μm to 13.1 μm as *Λ* increased from 200 nm to 1000 nm. The redshift of the resonance wavelength was the consequence of the decreasing reciprocal lattice vector of the periodical InAs strips. The reflection spectra of the resonator with varying *W* are shown in Figure 3c when *Λ* = 1000 nm, *H* = 100 nm, and *T* = 200 nm. It was observed that the resonant wavelength redshifted from 10.4 μm to 16.7 μm with increasing the *W* from 300 nm to 700 nm. This can be attributed to the increasing electromagnetic confinement induced by the strong coupling between adjacent InAs strips. As for the height *H* of the InAs strips, it was observed that the reflection dip of the resonator exhibited a blue-shift from 16.1 μm to 12.6 μm as *H* increased from 50 nm to 150 nm, as shown in Figure 3d. These results indicate that the resonance wavelength of the resonator can be tuned by the structural parameters of the InAs strips.

**Figure 4:**
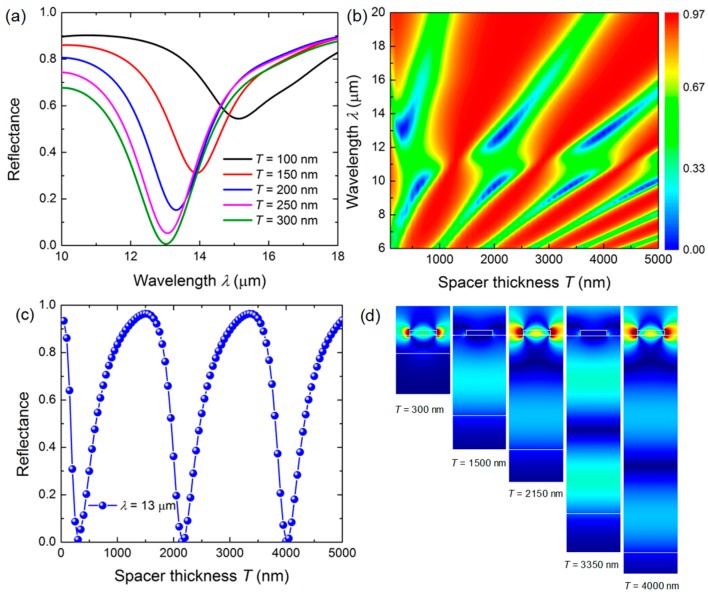
(**a**) Simulated reflection spectra with varying spacer thickness *T* of the undoped InAs. (**b**) Reflectance of the resonator mapping with various spacer thicknesses and the incident wavelengths. (**c**) The extracted reflectance with various *T* values when the wavelength of the incident light is 13 μm. (**d**) The electric field distribution when the spacer thickness *T* = 300 nm, 1500 nm, 2150 nm, 3350 nm, 4000 nm.

Then we explored the effect of the spacer thickness *T* on the spectral features of the resonator. It was found that the resonance absorption of the resonator could be modulated periodically by adjusting *T*. The reflection spectra with several sets of spacer thicknesses *T* are presented in Figure 4a. Obviously, one can see that the notch becomes narrower and deeper with the increasing *T*. When *T* = 300 nm, the reflectance becomes zero, indicating the complete absorption of the incident light by the resonator. To further investigate the modulation of *T* on the reflection spectra, the reflectance as a function of the spacer thickness *T* (in a wider range from 100 nm to 5000 m) and the wavelength *λ* is mapped in Figure 4b. The periodical variation tendency of the reflectance could be clearly observed with the varying spacer thickness. As an example, the reflectance with various *T* values when the wavelength of the incident light was 13 μm was extracted from Figure 4b and plotted in Figure 4c. One can estimate that the variation period of the reflectance is 1.85 μm. To explaine the periodical variation of reflectance, the electric field distribution when the spacer thickness *T* = 300 nm, 1500 nm, 2150 nm, 3350 nm and 4000 nm is displayed in Figure 4d. It shows that the field intensity aroud the strip can be modulated by tuning the spacer thickness, which is caused by the Fabry-Perot (F-P)–like interference formed between the top and the bottom highly doped InAs layer. This interference mode strongly coupled with the LSPP resonance mode, and led to an obvious avoided crossing shown in Figure 4b [27]. The corresponding variation period of the reflectance can be estimated by *T*_0_ = *λ*/2*n* (*T*_0_ = 1.85 μm when *λ* = 13 μm), which is in good agreement with the simulation results. Therefore, the resonance absorption of the resonator can be modulated periodically to achieve the maximum value by designing a suitable spacer thickness in the initial design.

### 3.2. SEIRAS of the Molecules

**Figure 5:**
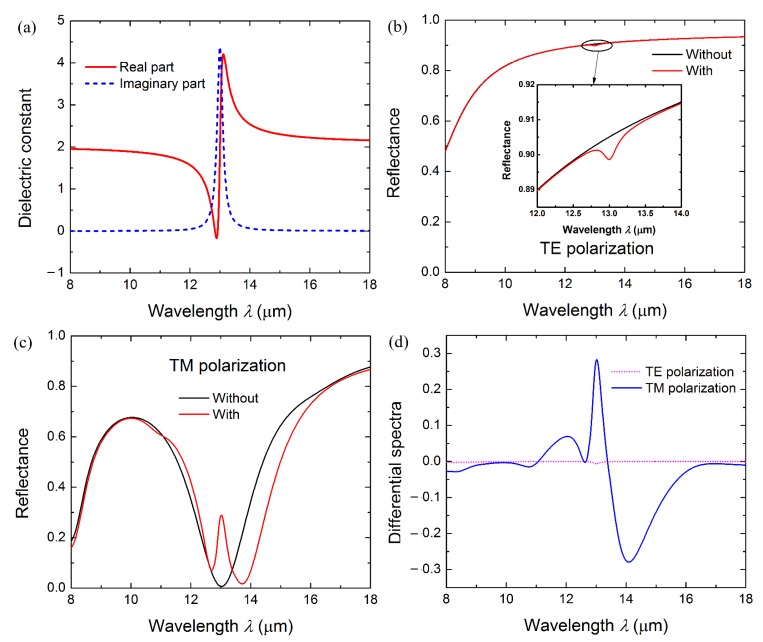
(**a**) The effective permittivity of the molecular film with one vibrational mode at 13.5 μm when *N_m_*= 5 × 10^23^ m^−3^, *γ_m_* = 2.5 × 10^12^ rad/s. (**b**) Reference reflection spectra of the resonator covered with (red) and without (black) 10-nm-thick molecular layer for TE-polarized light. (**c**) Reflection spectra of the resonator covered with (red) and without (black) 10-nm-thick molecular layer for TM-polarized light. (**d**) Reflectance difference (ΔR) of the resonator for TE- and TM-polarized light.

To explore the possible application of the proposed resonator in SEIRAS, we then considered the electromagnetic coupling between the plasmonic modes and infrared vibrational modes of molecules. A molecular film is assumed to be covered on the surface of the enhanced resonator. The resonator has structural parameters of *Λ* = 1000 nm, *W* = 500 nm, *H* = 100 nm and *T* = 300 nm. The molecular film has a vibrational mode at 770 cm^−1^ (13 μm, corresponding to the out-plane bending vibration of the C–H bond), the dielectric constant of which, calculated by Equation (3), is plotted in Figure 5a. To observe the pristine absorption of the molecular film without resonance enhancement, the reflection spectra of resonator covered with and without a molecular film for TE polarization light are presented in Figure 5b, respectively. A pristine weak vibration absorption signal of the molecular film also can be observed at the same wavelength position in the inset in Figure 5b, whereas it almost cannot be distinguished from the background curve, which is attributed to the weak interaction between the incident light and the ultra-thin molecular layer without a resonance effect for TE-polarized light in the mid-IR spectral range.

As a comparison, the reflection spectra of the plasmonic resonator covered with and without a molecular film for TM-polarized light are given in Figure 5c, respectively. An obvious reflection peak can be seen at the same frequency position of the molecular vibrational mode, which is due to the strong coupling between the vibrational mode of the molecular film and the TM resonance mode of the resonator when these two modes are overlapped. These results indicate that the plasmonic resonator can significantly enhance the absorption signal of the ultra-thin molecular layer.

To quantitatively evaluate the enhancement effect of the molecule absorption signal, the SEIRA enhancement factor is employed and calculated by Equation (4) [7]

(4) $$EF=\frac{\Delta R_{SEIRA}/\sigma_{SEIRA}}{\Delta R_{ref}/\sigma_{ref}}=\frac{\Delta R_{SEIRA}}{\Delta R_{ref}}\cdot \frac{\sigma_{ref}}{\sigma_{SEIRA}}$$

where Δ*R_ref_* is the enhanced signal of the resonator for the TE-polarized light, Δ*R_SEIRA_* is the enhanced signal of the resonator for the TM-polarized light, *σ_ref_* is the whole area of the substrate, and *σ_SEIRA_* is the effective enhancement area of the resonator. Here, the enhanced signal ΔR is defined as the differential spectra between the reflection curves of the resonator covered with and without a molecular film. The differential spectra of the resonator Δ*R_SEIRA_* and reference substrata Δ*R_ref_* are calculated from Figure 5b,c and compared in Figure 5d, respectively. The Δ*R_SEIRA_* of the resonator is determined to be about 28.2%, which is 94 folds larger than that (only 0.3%) of the reference substrate. Meanwhile, it is also necessary to consider the fact that the observed enhanced absorption signal is due to the molecules at the close vicinity of the InAs strip edges, where high-intensity plasmons are excited (Figure 3a). In each period, the fraction of the active area *σ_SEIRA_* with intense near-fields compared to the whole area *σ_ref_* is around 2*H*/*Λ* [2]. Considering these factors together, the SEIRA enhancement factor of the resonator is about 470 at 770 cm^−1^ (13 μm) for molecular film, which further indicates the resonator as a promising SEIRAS substrate.

## 4. Conclusions

In summary, an all-semiconductor plasmonic resonator is proposed to enhance the infrared absorption of the molecules. It is found that highly doped InAs strips support surface plasmon resonance that can be tuned by the structural parameters of patterned InAs strips, and the resonance absorption of the resonator can be modulated periodically by adjusting the spacer thickness. The enhanced electric field gives a SEIRA enhancement factor of more than 470 for a thin molecular film with a vibration frequency around 770 cm^−1^ (13 μm) when the resonance of the resonator and the absorption peak of the molecules are overlapped. Our study opens an avenue for engineering semiconductor optics devices for SEIRAS.
